# 2,4-Dichloro-6-methoxy­quinoline

**DOI:** 10.1107/S1600536809002402

**Published:** 2009-01-23

**Authors:** R. Subashini, Venkatesha R. Hathwar, P. Manivel, K. Prabakaran, F. Nawaz Khan

**Affiliations:** a Organic Chemistry Division, School of Science and Humanities, VIT University, Vellore 632 014, Tamil Nadu, India; b Solid State and Structural Chemistry Unit, Indian Institute of Science, Bangalore 560 012, Karnataka, India

## Abstract

The title compound, C_10_H_7_Cl_2_NO, features a planar mol­ecule, excluding the methyl H atoms [maximum deviation = 0.0385 (1) Å]. The crystal packing is stabilized by π–π stacking inter­actions across inversion centres [centroid-to-centroid distance = 3.736 (3) Å].

## Related literature

For general background, see: Fournet *et al.* (1981 ▶) and references cited therein; Towers *et al.* (1981 ▶); Biavatti *et al.* (2002 ▶); McCormick *et al.* (1996 ▶); Ziegler & Gelfert, (1959 ▶). For related crystal structures, see: Somvanshi *et al.* (2008 ▶).
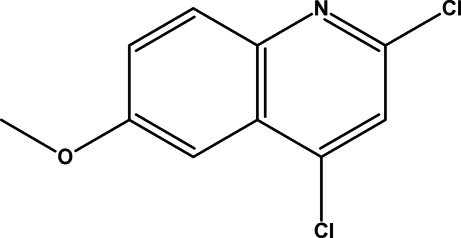


## Experimental

### 

#### Crystal data

C_10_H_7_Cl_2_NO
*M* *_r_* = 228.07
Triclinic, 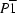

*a* = 7.431 (2) Å
*b* = 8.889 (2) Å
*c* = 9.083 (4) Å
α = 116.660 (19)°
β = 102.301 (2)°
γ = 104.150 (14)°
*V* = 482.5 (3) Å^3^
*Z* = 2
Mo *K*α radiation
μ = 0.63 mm^−1^
*T* = 290 (2) K
0.25 × 0.18 × 0.15 mm

#### Data collection

Bruker SMART CCD area-detector diffractometer
Absorption correction: multi-scan (*SADABS*; Sheldrick, 1996 ▶) *T*_min_ = 0.811, *T*_max_ = 0.909
5720 measured reflections
1782 independent reflections
1272 reflections with *I* > 2σ(*I*)
*R*_int_ = 0.054

#### Refinement

*R*[*F* ^2^ > 2σ(*F* ^2^)] = 0.065
*wR*(*F* ^2^) = 0.197
*S* = 1.15
1782 reflections
128 parameters
H-atom parameters constrained
Δρ_max_ = 0.38 e Å^−3^
Δρ_min_ = −0.64 e Å^−3^

### 

Data collection: *SMART* (Bruker, 2004 ▶); cell refinement: *SAINT* (Bruker, 2004 ▶); data reduction: *SAINT*; program(s) used to solve structure: *SHELXS97* (Sheldrick, 2008 ▶); program(s) used to refine structure: *SHELXL97* (Sheldrick, 2008 ▶); molecular graphics: *ORTEP-3* (Farrugia, 1999 ▶) and *CAMERON* (Watkin *et al.*, 1993 ▶); software used to prepare material for publication: *PLATON* (Spek, 2003 ▶).

## Supplementary Material

Crystal structure: contains datablocks global, I. DOI: 10.1107/S1600536809002402/bt2852sup1.cif

Structure factors: contains datablocks I. DOI: 10.1107/S1600536809002402/bt2852Isup2.hkl

Additional supplementary materials:  crystallographic information; 3D view; checkCIF report

## Figures and Tables

**Fig. 1.**
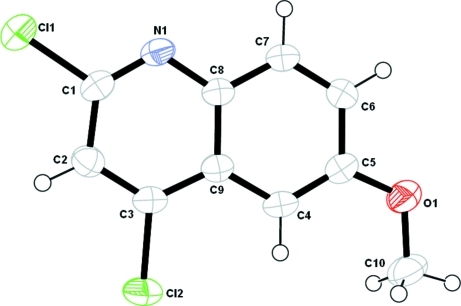
ORTEP diagram of the title compound with 50% probability displacement ellipsoids.

**Fig. 2.**
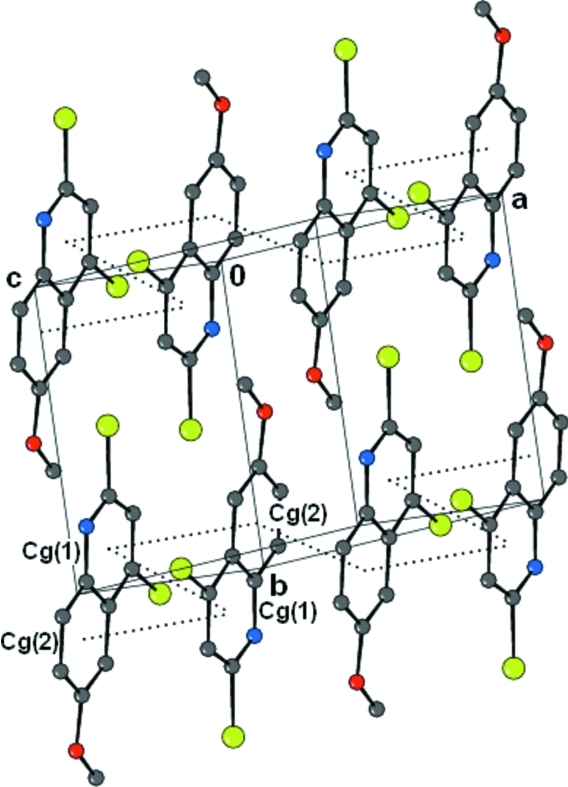
The crystal packing diagram of the title compound. The dotted lines indicate π–π interactions. All H atoms have been omitted for clarity.

## supplementary crystallographic information

### Comment

A wide range of medicinal properties have already been identified in compounds containing the quinoline ring system including antiprotozoal (Fournet *et al.*, 1981), antibacterial (Towers *et al.*, 1981), antifungal (Biavatti *et al.*, 2002) and antiviral activities (McCormick *et al.*, 1996). Reaction of aniline with malonic acid in an excess of phosphorus oxychloride at reflux to give 2,4-dichloroquinoline was first reported by Ziegler & Gelfert (1959). A similar derivative of quinoline was synthesized from the mixture of *p*-toluidine and malonic acid in a one-pot reaction from an aryl amine, malonic acid and phosphorous oxychloride and its cytotoxicity has been reported (Somvanshi & Subashini *et al.*, 2008). In continuous of our work, crystal structure of another derivative is reported in this paper.

The crystal packing is stabilized by intermolecular π–π [*Cg*1···*Cg*1 and *Cg*2···*Cg*2] stacking interactions with shortest perpendicular distances between isochinoline groups of 3.470 Å and 3.497 Å, the slippages between these ring systems are 1.283 Å and 1.178 Å, the distances between the centroids of the six-membered carbon rings are 3.700 (3) Å and 3.690 (3) Å with the symmetry code (2 - *x*, -*y*, 1 - *z*) and (1 - *x*, -*y*, 1 - *z*), respectively. Further, another intermolecular π–π [*Cg*1···*Cg*2] stacking interactions with a shortest perpendicular distance of 3.476 Å between the two rings and the distance between the centroids of the six-membered carbon rings is 3.736 (3) Å with the symmetry code (2 - *x*, -*y*, -*z*). *Cg*1 and *Cg*2 are the centroids of N1—C1—C2—C3—C4—C8—C9 ring and C4–C9 ring respectively.

### Experimental

*p*-Anisidine (10 mmol) and malonic acid (15 mmol) were heated under reflux in phosphorus oxychloride (20 ml), with stirring, for 5 h. The mixture was cooled, poured into crushed ice with vigorous stirring and then made alkaline with 5 *M* sodium hydroxide. Filtration gave the crude product as a brown solid. A Column chromatography (95:5 hexane–EtOAc) yielded the pure dichloroquinoline as off-white needles

### Refinement

All the H atoms were positioned geometrically and refined using a riding model [C—H = 0.97 Å and *U*_iso_(H) = 1.5*U*_eq_(C) for methyl and C—H = 0.93 Å and *U*_iso_(H) = 1.2*U*_eq_(C) for all other H atoms.

### Crystal data

**Table:**

C_10_H_7_Cl_2_NO	*Z* = 2
*M**_r_* = 228.07	*F*(000) = 232
Triclinic, *P*1	*D*_x_ = 1.570 Mg m^−^^3^
Hall symbol: -P 1	Mo *K*α radiation, λ = 0.71073 Å
*a* = 7.431 (2) Å	Cell parameters from 856 reflections
*b* = 8.889 (2) Å	θ = 1.9–20.7°
*c* = 9.083 (4) Å	µ = 0.63 mm^−^^1^
α = 116.660 (19)°	*T* = 290 K
β = 102.301 (2)°	Block, colourless
γ = 104.150 (14)°	0.25 × 0.18 × 0.15 mm
*V* = 482.5 (3) Å^3^	

### Data collection

**Table:**

Bruker SMART CCD area-detector diffractometer	1782 independent reflections
Radiation source: fine-focus sealed tube	1272 reflections with *I* > 2σ(*I*)
graphite	*R*_int_ = 0.054
φ and ω scans	θ_max_ = 25.5°, θ_min_ = 2.7°
Absorption correction: multi-scan (*SADABS*; Sheldrick, 1996)	*h* = −5→8
*T*_min_ = 0.811, *T*_max_ = 0.909	*k* = −10→10
5720 measured reflections	*l* = −11→11

### Refinement

**Table:**

Refinement on *F*^2^	Primary atom site location: structure-invariant direct methods
Least-squares matrix: full	Secondary atom site location: difference Fourier map
*R*[*F*^2^ > 2σ(*F*^2^)] = 0.065	Hydrogen site location: inferred from neighbouring sites
*wR*(*F*^2^) = 0.197	H-atom parameters constrained
*S* = 1.15	*w* = 1/[σ^2^(*F*_o_^2^) + (0.0844*P*)^2^ + 0.6982*P*] where *P* = (*F*_o_^2^ + 2*F*_c_^2^)/3
1782 reflections	(Δ/σ)_max_ < 0.001
128 parameters	Δρ_max_ = 0.38 e Å^−^^3^
0 restraints	Δρ_min_ = −0.64 e Å^−^^3^

### Special details

**Table:**

Geometry. All e.s.d.'s (except the e.s.d. in the dihedral angle between two l.s. planes) are estimated using the full covariance matrix. The cell e.s.d.'s are taken into account individually in the estimation of e.s.d.'s in distances, angles and torsion angles; correlations between e.s.d.'s in cell parameters are only used when they are defined by crystal symmetry. An approximate (isotropic) treatment of cell e.s.d.'s is used for estimating e.s.d.'s involving l.s. planes.
Refinement. Refinement of *F*^2^ against ALL reflections. The weighted *R*-factor *wR* and goodness of fit *S* are based on *F*^2^, conventional *R*-factors *R* are based on *F*, with *F* set to zero for negative *F*^2^. The threshold expression of *F*^2^ > σ(*F*^2^) is used only for calculating *R*-factors(gt) *etc*. and is not relevant to the choice of reflections for refinement. *R*-factors based on *F*^2^ are statistically about twice as large as those based on *F*, and *R*- factors based on ALL data will be even larger.

### Fractional atomic coordinates and isotropic or equivalent isotropic displacement parameters (Å^2^)

**Table:**

	*x*	*y*	*z*	*U*_iso_*/*U*_eq_	
Cl1	0.3126 (2)	1.55448 (15)	0.76004 (16)	0.0555 (4)	
Cl2	0.2701 (2)	0.98994 (16)	0.84707 (16)	0.0592 (5)	
N1	0.2689 (5)	1.2373 (5)	0.5081 (5)	0.0400 (9)	
O1	0.2020 (5)	0.5198 (4)	0.1887 (4)	0.0517 (8)	
C1	0.2837 (6)	1.3262 (5)	0.6714 (6)	0.0383 (10)	
C2	0.2849 (6)	1.2594 (5)	0.7862 (5)	0.0382 (9)	
H2	0.2972	1.3312	0.9031	0.046*	
C3	0.2666 (6)	1.0805 (6)	0.7130 (5)	0.0378 (9)	
C4	0.2323 (6)	0.7880 (5)	0.4545 (6)	0.0380 (9)	
H4	0.2318	0.7305	0.5184	0.046*	
C5	0.2168 (6)	0.6950 (5)	0.2814 (6)	0.0392 (10)	
C6	0.2134 (7)	0.7815 (6)	0.1824 (6)	0.0422 (10)	
H6	0.1994	0.7169	0.0641	0.051*	
C7	0.2302 (7)	0.9572 (6)	0.2591 (6)	0.0421 (10)	
H7	0.2294	1.0119	0.1926	0.051*	
C8	0.2489 (6)	1.0586 (5)	0.4363 (5)	0.0350 (9)	
C9	0.2491 (6)	0.9716 (5)	0.5361 (5)	0.0344 (9)	
C10	0.2067 (8)	0.4245 (7)	0.2807 (7)	0.0573 (13)	
H10A	0.3330	0.4855	0.3778	0.086*	
H10B	0.1897	0.3014	0.2006	0.086*	
H10C	0.1007	0.4228	0.3254	0.086*	

### Atomic displacement parameters (Å^2^)

**Table:**

	*U*^11^	*U*^22^	*U*^33^	*U*^12^	*U*^13^	*U*^23^
Cl1	0.0789 (9)	0.0407 (6)	0.0592 (8)	0.0310 (6)	0.0316 (6)	0.0293 (6)
Cl2	0.0927 (11)	0.0533 (7)	0.0488 (7)	0.0298 (7)	0.0288 (6)	0.0383 (6)
N1	0.049 (2)	0.0396 (19)	0.046 (2)	0.0226 (17)	0.0195 (17)	0.0300 (17)
O1	0.070 (2)	0.0391 (16)	0.0490 (18)	0.0252 (16)	0.0202 (16)	0.0246 (15)
C1	0.038 (2)	0.035 (2)	0.047 (2)	0.0168 (18)	0.0141 (19)	0.0248 (19)
C2	0.038 (2)	0.044 (2)	0.038 (2)	0.0176 (19)	0.0164 (18)	0.0234 (19)
C3	0.040 (2)	0.042 (2)	0.041 (2)	0.0169 (18)	0.0150 (18)	0.0284 (19)
C4	0.039 (2)	0.039 (2)	0.044 (2)	0.0151 (18)	0.0138 (19)	0.0295 (19)
C5	0.039 (2)	0.035 (2)	0.045 (2)	0.0148 (18)	0.0130 (19)	0.0232 (19)
C6	0.053 (3)	0.045 (2)	0.036 (2)	0.023 (2)	0.0185 (19)	0.0234 (19)
C7	0.055 (3)	0.044 (2)	0.042 (2)	0.025 (2)	0.020 (2)	0.030 (2)
C8	0.036 (2)	0.037 (2)	0.038 (2)	0.0151 (17)	0.0127 (17)	0.0239 (18)
C9	0.032 (2)	0.036 (2)	0.039 (2)	0.0127 (17)	0.0127 (17)	0.0235 (18)
C10	0.070 (3)	0.048 (3)	0.074 (3)	0.028 (2)	0.030 (3)	0.043 (3)

### Geometric parameters (Å, °)

**Table:**

Cl1—C1	1.749 (4)	C4—C9	1.415 (5)
Cl2—C3	1.734 (4)	C4—H4	0.9300
N1—C1	1.293 (5)	C5—C6	1.422 (6)
N1—C8	1.372 (5)	C6—C7	1.352 (6)
O1—C5	1.359 (5)	C6—H6	0.9300
O1—C10	1.433 (5)	C7—C8	1.402 (6)
C1—C2	1.412 (5)	C7—H7	0.9300
C2—C3	1.377 (6)	C8—C9	1.432 (5)
C2—H2	0.9300	C10—H10A	0.9600
C3—C9	1.411 (6)	C10—H10B	0.9600
C4—C5	1.370 (6)	C10—H10C	0.9600
			
C1—N1—C8	117.4 (3)	C7—C6—H6	119.8
C5—O1—C10	117.2 (4)	C5—C6—H6	119.8
N1—C1—C2	126.7 (4)	C6—C7—C8	121.6 (4)
N1—C1—Cl1	116.3 (3)	C6—C7—H7	119.2
C2—C1—Cl1	117.0 (3)	C8—C7—H7	119.2
C3—C2—C1	115.3 (4)	N1—C8—C7	119.1 (3)
C3—C2—H2	122.3	N1—C8—C9	122.6 (4)
C1—C2—H2	122.3	C7—C8—C9	118.3 (4)
C2—C3—C9	122.4 (3)	C3—C9—C4	125.0 (4)
C2—C3—Cl2	117.9 (3)	C3—C9—C8	115.5 (3)
C9—C3—Cl2	119.6 (3)	C4—C9—C8	119.5 (4)
C5—C4—C9	120.1 (4)	O1—C10—H10A	109.5
C5—C4—H4	120.0	O1—C10—H10B	109.5
C9—C4—H4	120.0	H10A—C10—H10B	109.5
O1—C5—C4	125.8 (4)	O1—C10—H10C	109.5
O1—C5—C6	114.1 (4)	H10A—C10—H10C	109.5
C4—C5—C6	120.0 (4)	H10B—C10—H10C	109.5
C7—C6—C5	120.5 (4)		
			
C8—N1—C1—C2	1.2 (6)	C1—N1—C8—C9	−1.6 (6)
C8—N1—C1—Cl1	179.1 (3)	C6—C7—C8—N1	178.9 (4)
N1—C1—C2—C3	−0.6 (6)	C6—C7—C8—C9	−0.2 (7)
Cl1—C1—C2—C3	−178.5 (3)	C2—C3—C9—C4	179.2 (4)
C1—C2—C3—C9	0.4 (6)	Cl2—C3—C9—C4	0.4 (6)
C1—C2—C3—Cl2	179.2 (3)	C2—C3—C9—C8	−0.8 (6)
C10—O1—C5—C4	−1.2 (6)	Cl2—C3—C9—C8	−179.6 (3)
C10—O1—C5—C6	179.3 (4)	C5—C4—C9—C3	−179.8 (4)
C9—C4—C5—O1	179.3 (4)	C5—C4—C9—C8	0.3 (6)
C9—C4—C5—C6	−1.3 (6)	N1—C8—C9—C3	1.5 (6)
O1—C5—C6—C7	−178.9 (4)	C7—C8—C9—C3	−179.5 (4)
C4—C5—C6—C7	1.6 (7)	N1—C8—C9—C4	−178.6 (4)
C5—C6—C7—C8	−0.8 (7)	C7—C8—C9—C4	0.5 (6)
C1—N1—C8—C7	179.3 (4)		

## References

[bb1] Biavatti, M. W., Vieira, P. C., da Silva, M. F. G. F., Fernandes, J. B., Victor, S. R., Pagnocca, F. C., Albuquerque, S., Caracelli, I. & Zukerman-Schpector, J. (2002). *J. Braz. Chem. Soc.* **13**, 66–70.

[bb2] Bruker (2004). *SMART* and *SAINT* Bruker AXS Inc., Madison, Wisconsin, USA.

[bb3] Farrugia, L. J. (1999). *J. Appl. Cryst.* **32**, 837–838.

[bb4] Fournet, A., Barrios, A. A., Munioz, V., Hocquemiller, R., Cave, A. & Bruneton, J. (1981). *J. Antimicrob. Agents Chemother.* **37**, 859–863. 10.1128/aac.37.4.859 PMC187784 8494383

[bb5] McCormick, J. L., McKee, T. C., Cardellina, J. H. & Boyd, M. R. (1996). *J. Nat. Prod.* **59**, 469–471. 10.1021/np960250m 8778237

[bb6] Sheldrick, G. M. (1996). *SADABS* University of Göttingen, Germany.

[bb7] Sheldrick, G. M. (2008). *Acta Cryst.* A**64**, 112–122. 10.1107/S0108767307043930 18156677

[bb8] Somvanshi, R. K., Subashini, R., Dhanasekaran, V., Arulprakash, G., Das, S. N. & Dey, S. (2008). *J. Chem. Crystallogr.* 38, 381–386.

[bb9] Spek, A. L. (2003). *J. Appl. Cryst.* **36**, 7–13.

[bb10] Towers, G. H. N., Grahanm, E. A., Spenser, I. D. & Abramowski, Z. (1981). *Planta Med.* **41**, 136–142. 10.1055/s-2007-971690 7232551

[bb11] Watkin, D. J., Pearce, L. & Prout, C. K. (1993). *CAMERON* Chemical Crystallography Laboratory, University of Oxford, England.

[bb12] Ziegler, E. & Gelfert, K. (1959). *Monatsh. Chem.* **90**, 822–826.

